# Influence of solar extreme ultraviolet radiations on artificial very-low-frequency waves in near-earth space

**DOI:** 10.1038/s41598-025-14293-5

**Published:** 2025-08-13

**Authors:** K. X. Cheng, L. Y. Li, L. Yang, J. B. Cao, J. Yu, S. F. Zhao

**Affiliations:** 1 School of Space and Earth Sciences, Beihang University, Beijing, China; 2 Key Laboratory of Space Environment Monitoring, Information Processing of MIIT, Beijing, China; 3 Planetary Environmental Astrobiological Research Laboratory (PEARL), School of Atmospheric Sciences, Sun Yat-sen University, Zhuhai, China; 4 State Key Laboratory of Space Weather, National Space Science Center, Chinese Academy of Sciences, Beijing, China

**Keywords:** Space physics, Astronomy and planetary science

## Abstract

Ground-based very low frequency (VLF) transmitter waves (3 – 30 kHz) can cause the precipitation loss of high-energy electrons in Earth’s radiation belts. Although the propagation and attenuation of artificial VLF waves have been studied for more than half a century, it is not clear whether solar extreme ultraviolet (EUV) radiations can modify the VLF wave intensity in inner radiation belt and slot region (*L* ~ 1.1 – 3R_E_). Here, by analyzing satellite observations and quantitative calculations, we find that the enhanced solar EUV radiations cause global attenuation of the artificial VLF waves radiated from low-latitude transmitters (λ < 44.2° or* L* < 1.8 R_E_), whereas those waves radiated from middle-latitude transmitters (λ > 44.2° or *L* > 1.8 R_E_) weaken slightly around noon. Under high solar EUV radiations, the large attenuation of artificial VLF waves in the low L region is due to enhanced collisional damping of ionospheric plasmas at low latitudes.

## Introduction

Ground-based VLF transmitter waves have widely used for long distance communications with surface ships and submerged submarines^1^ and for early geo-location^2^. A fraction of artificial VLF waves can leak into Earth’s ionosphere (*h* ~ 60–1000 km)^3–5^ and heat ionospheric cold plasmas^6^^,^^7^. High-power VLF waves propagate even into inner radiation belt (*L* ~ 1.1–2 R_E_) and slot region (*L* ~ 2–3 R_E_)^8–13^ and cause the precipitation of high-energy radiation belt electrons^14–23^. Artificial VLF waves sometimes coexist with natural plasmaspheric hiss (~ 0.05–2 kHz)^24,25^ and lightning-generated whistlers (~ 0.1–40 kHz)^26,27^ in the near-earth space, and they resonate with different-energy electrons and eventually result in the loss of more electrons^28–31^.

Since artificial VLF waves have significant influences on the near-earth space environments, their propagation and attenuation have been an interesting topic in space physics. Full-wave modeling indicate that the trans-ionospheric attenuation of VLF waves radiated from low-latitude transmitters is larger than that radiated from middle-latitude transmitters^32–34^, and the non-ducted VLF waves experience larger attenuations than the ducted VLF waves^35^. Satellite observations indicate that artificial VLF waves mostly propagate in non-ducted modes in the region of L < 1.8 R_E_, whereas for L > 1.8 R_E_, ducted propagation becomes the dominant mechanism^36,37^. The artificial VLF waves in the near-earth space have significantly diurnal and seasonal variations^38,39^. Moreover, the VLF radio signals measured on the ground have also remarkable responses to solar X-ray flares that modify the D region (~ 60–90 km) of ionosphere^40,41^.

Besides solar X-ray flares, solar EUV radiations can also cause the ionization of neutral atmospheric compositions in the F region (> 160 km) of ionosphere^42,43^. However, it is not clear whether the change in solar EUV radiations can modify the intensity of artificial VLF waves in the near-earth space. Here, with Van Allen Probe A observations and theoretical calculations, we revealed the different responses of artificial VLF waves from low- and middle-latitude transmitters in the near-earth space to the increase in solar EUV radiations.

## Results

The power spectral density (P_E_) of artificial VLF waves is recorded by the Electric and Magnetic Field Instrument Suite and Integrated Science (EMFISIS)^44^ on board the Probe A near the magnetic equator below 20 degrees under different solar EUV radiations. The intensity of solar EUV radiations is indicated by the daily averaged 10.7 cm solar radio flux (F10.7)^45^. The elliptical orbit (perigee ~ 1.1R_E_, and apogee ~ 6.5R_E_) of Probe A covers most regions of the inner and outer radiation belts (*L* ~ 1.1 ~ 7R_E_). Probe A at different L shells can observe the artificial VLF waves radiated from ground-based transmitters at different latitudes.

**Fig. 1:**
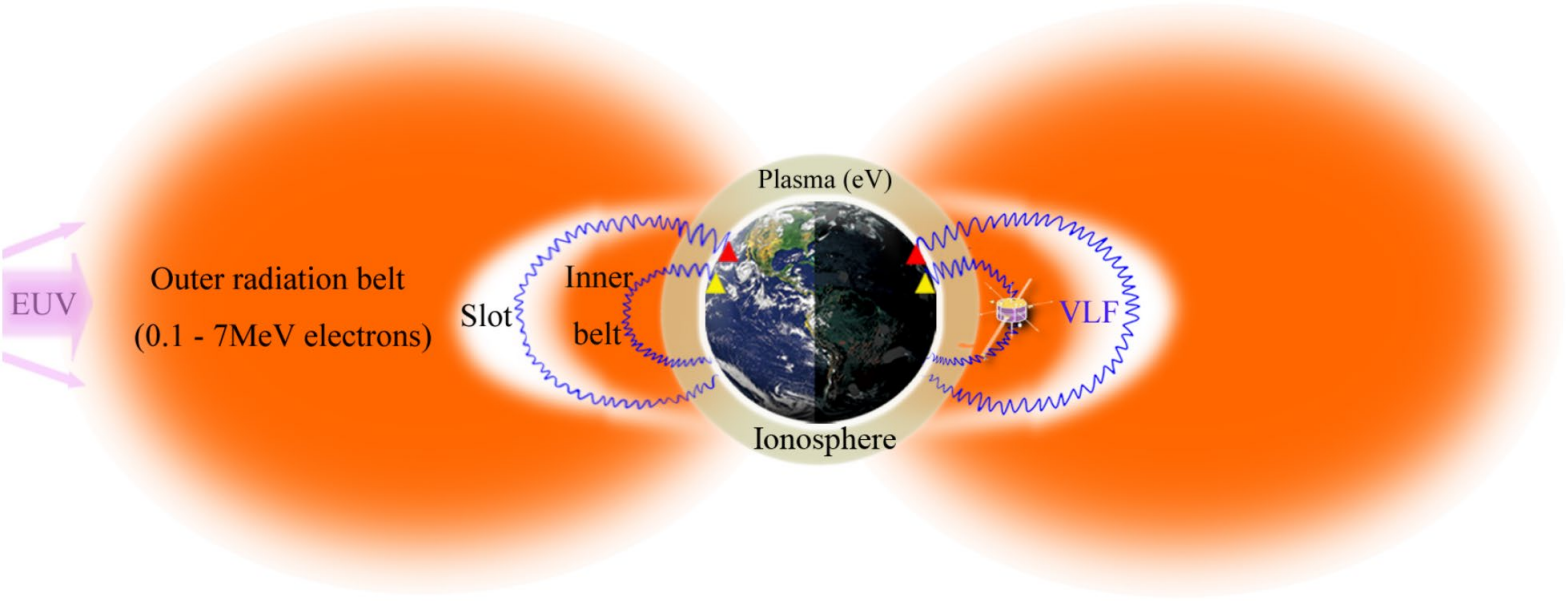
Schematic illustration of the propagation path of artificial VLF waves in near-earth space. Yellow and red triangles roughly denote the low- and middle-latitude VLF transmitters, respectively.

Figure 1 roughly illustrates the propagation path of ground-based VLF transmitter waves (blue wavy curves) in the near-earth space. The upward propagating VLF waves experience collision damping by cold plasmas in the ionosphere (*h* ~ 60–1000 km). A little fraction of VLF waves radiated from low-latitude transmitters (yellow triangles) propagate through the low-latitude ionosphere and eventually enter the inner radiation belt (*L* ~ 1.1–2 R_E_), while others from middle-latitude transmitters (red triangles) propagate through the middle-latitude ionosphere and eventually enter the slot region (*L* ~ 2–3 R_E_).

**Fig. 2:**
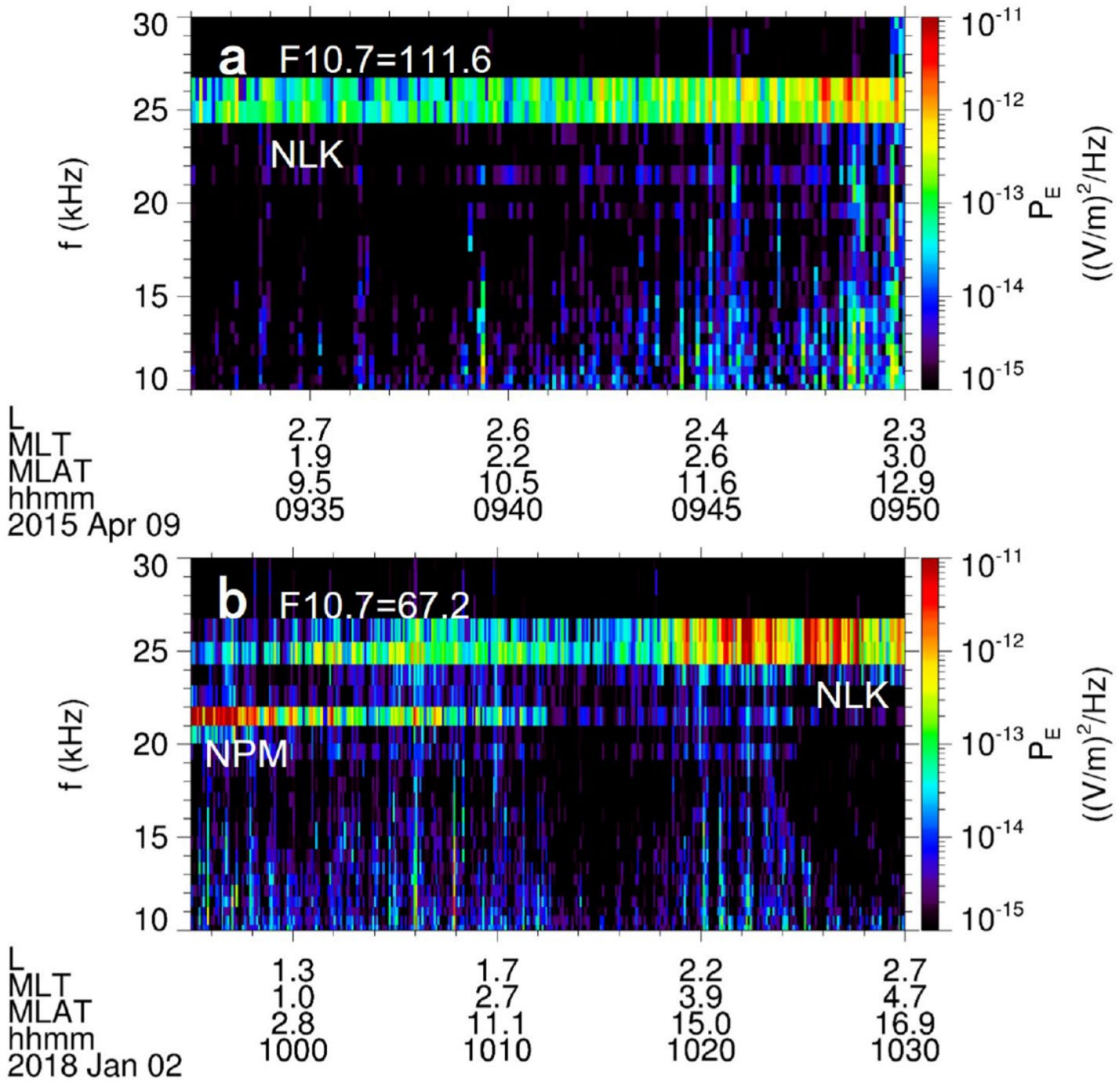
The electric field power spectral density of artificial VLF waves observed by Probe A in the inner radiation belt and slot region. L is the distance (in R_E_) from the point where a magnetic field line crosses the equatorial plane to the center of the Earth. MLT is magnetic local time (hour), and MLAT is magnetic latitude (degree).

Figures 2a and 2b display the power spectral density of electric field observed by Probe A in 2015 and 2018 under different F10.7 conditions. The power spectral density of artificial VLF waves is larger than 10^−13^ (V/m)^2^/Hz in the frequency ranges of 20–22 kHz and 24–27 kHz. The spatial coverage range of artificial VLF waves is typically ~ 1000–3000 km (~ 0.16–0.47R_E_)^8^. Based on the wave frequency and transmitter location (L) in Table 1, we can determine the transmitter latitude (λ) of VLF waves. Therefore, the VLF waves (*f* ~ 21–22kHz) in the region of *L* ~ 1.13 ± 0.47 R_E_ are radiated from the NPM transmitter (*f* ~ 21.4 kHz, *L* ~ 1.13 R_E_), while those waves (*f* ~ 24–26kHz) in the region of *L* ~ 2.78 ± 0.47R_E_ are radiated from the NLK transmitter (*f* ~ 24.8 kHz,* L* ~ 2.78 R_E_). Although the NLK transmitter power (250 kW) is fixed, the power spectral density of the VLF waves radiated from NLK is larger in low F10.7 year (2018) than in high F10.7 year (2015) in the near-earth space.

**Table 1:** List of ground-based VLF transmitters. L is calculated with the IGRF-14 magnetic field model in 2018.

Transmitters	Geographic Latitude λ (°)	L (R_E_)	Frequency (kHz)	Power (kW)
NPM	21.42N	1.13	21.4	600
JJI	32.09N	1.15	22.2	100
unid25	34.58N	1.21	25.0	250
TBB	37.41N	1.37	26.7	~ 100
ICV	40.92N	1.48	20.3	50
HWU	46.71N	1.82	18.3/19.1/21.75/22.6	200
FTA2	48.54N	1.97	20.9	~ 50
DHO38	53.09N	2.39	23.4	300
GVT	54.73N	2.65	22.1	100
NAA	44.64N	2.71	24.0	1000
NLK	48.20	2.79	24.8	250
NML	46.37N	3.10	25.2	250

Figure 2 indicates that the power spectral densities of the narrow-band VLF waves (horizontal stripes) are mostly larger than those of the wide-band noise (vertical stripes) or lightning-generated whistlers (LGWs ~ 0.1–40 kHz). The frequencies of effective LGWs (P_E_ ≥ 10^−13^ (V/m)^2^/Hz) are mostly lower than 20 kHz^26,27^. Therefore, we only selected the 20.05–26.74 kHz waves and set a threshold power (~ 10^−13^ (V/m)^2^/Hz) to eliminate background noise and LGWs. As indicated by Table 1, the selected VLF waves (20.05–26.74 kHz) are radiated by the ground-based transmitters in the northern hemisphere. The selected band effectively excludes the VLF waves from the NWC transmitter (*f* ~ 19.8 kHz, λ ~ 21.82°S) in the southern hemisphere, ensuring that the average intensity of VLF waves under different solar EUV radiations is not affected by the oppositely seasonal variations in both hemispheres.

To avoid the influence of geomagnetic activities, the selected VLF waves (*f* ~ 20.05–26.74 kHz, and P_E_ ≥ 10^−13^ (V/m)^2^/Hz) came from the Probe A observations only during geomagnetically quiet times (AE < 200 nT). The statistical period is from 7 September 2012 to 30 June 2019. According to the typical boundary (L ~ 1.8R_E_) of ducted and non-ducted VLF waves^38,39^, the selected waves are divided into two groups inside and outside this boundary. In order to simplify the description, we approximate the region of L < 1.8 R_E_ (non-ducted waves) as the inner radiation belt, and the region L > 1.8 R_E_ (ducted waves) as the slot region. The amplitude of selected VLF waves (E_w_) is calculated by Equation 1 in the Method Section. Finally, we averaged the wave amplitudes in each grid (0.1L×1MLT) under different levels of solar EUV radiations (F10.7 < 75, 70 ≤ F10.7 < 110, F10.7 ≥ 110 (in 10^−22^ Wm^−2^ Hz^−1^)). In addition, we estimated the number density (*N*_e_) of ionospheric cold electrons with the International Reference Ionosphere model (IRI-2020) under each level of solar EUV radiations.

**Fig. 3:**
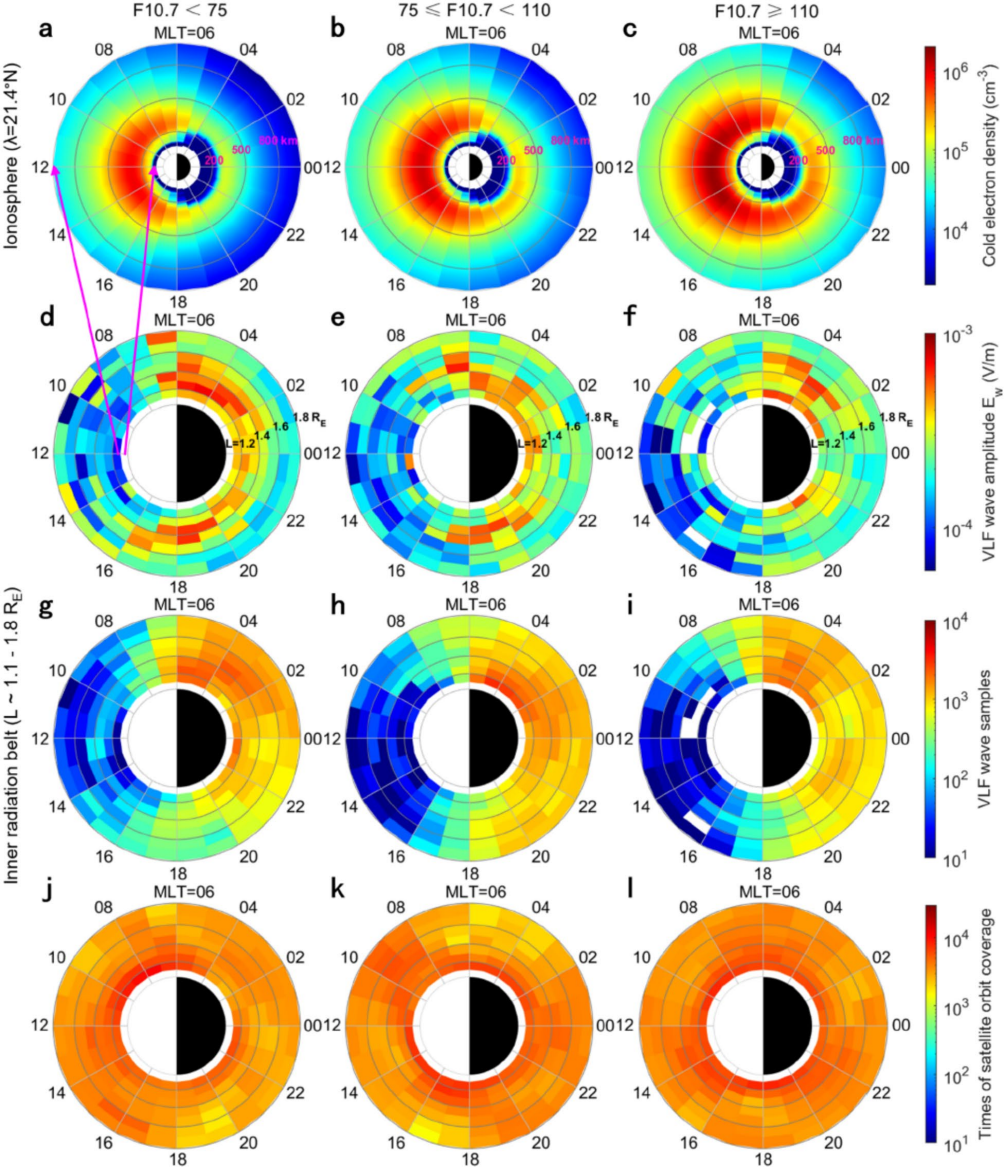
(**a**-**c**) The height (60–1000 km) and MLT distributions of ionospheric electron density at low latitudes under different solar EUV radiations (F10.7).* N*_e_ is from the IRI-2020 model at λ=21.4°N. (d – i) The radial (L) and MLT distributions of VLF wave amplitudes and samples (20.05–26.74 kHz) measured by Probe A in the inner radiation belt region (L ~ 1.1–1.8 R_E_). (j – l) The times of Probe A crossings during statistical period.

Figures 3a – c show the height and MLT distributions of the ionospheric electron density at low latitudes of the northern hemisphere (λ=21.4°N) under different solar EUV radiations. The density of ionospheric electrons is generally higher on the dayside (MLT ~ 6–18 h) than the nightside (MLT ~ 18–6 h) at the same altitude, as neutral atmospheric compositions experience photoionization on the dayside under sunlight irradiations. As indicated by Figure 1, most regions of the low-latitude ionosphere can absorb vertical solar EUV radiations. Thus, the ionospheric electron density at low latitudes (λ=21.4°N) remarkably increases when solar EUV radiations (F10.7) intensify from 75 to 110. The increase of dayside electron density is significant in the whole D, E and F regions (60–1000 km), whereas the nightside electron density increases mainly in the F region above 200 km. Although the enhanced solar EUV radiations weaken the day-night asymmetry of ionospheric electron density, the electron density is still the lowest on the dawnside (MLT = 02–05 h) under different solar EUV radiations.

The day-night asymmetry of ionospheric electron density results in the opposite variation of artificial VLF waves in the inner radiation belt region (L < 1.8). Figures 3d – i display the amplitudes and samples of global VLF waves measured by Probe A under different solar EUV radiations. According to Table 1, the artificial VLF waves (20.05–26.74 kHz) in the inner radiation belt region are mostly radiated from low-latitude transmitters (λ ≤ 44.2°N) in the northern hemisphere. Under low solar EUV radiations (F10.7 < 75), the VLF wave amplitudes in the daytime sector (MLT ~ 07–16 h) are obviously lower than those at other MLTs (Figure 3d), and the MLT distribution of the samples of selected VLF waves has similar day-night asymmetry (Figure 3g). The day-night asymmetric distributions of artificial VLF waves are different from the relatively uniform spatial coverage of Probe A orbit on the L-MLT plane, as indicated by Figures 3 j – l. When solar EUV radiations gradually intensify (F10.7 increases from 75 to 110), the amplitudes and samples of VLF waves decrease remarkably on the whole dayside (MLT ~ 06–18 h in Figures 3d - 3i). The decay of the VLF wave amplitudes extends to most regions in nighttime (MLT ~ 18–02 h) under high solar EUV radiations (F10.7 ≥ 110). However, the VLF wave amplitudes are still maximum in the dawn sector (MLT ~ 02–06 h) with a minimum electron density.

**Fig. 4:**
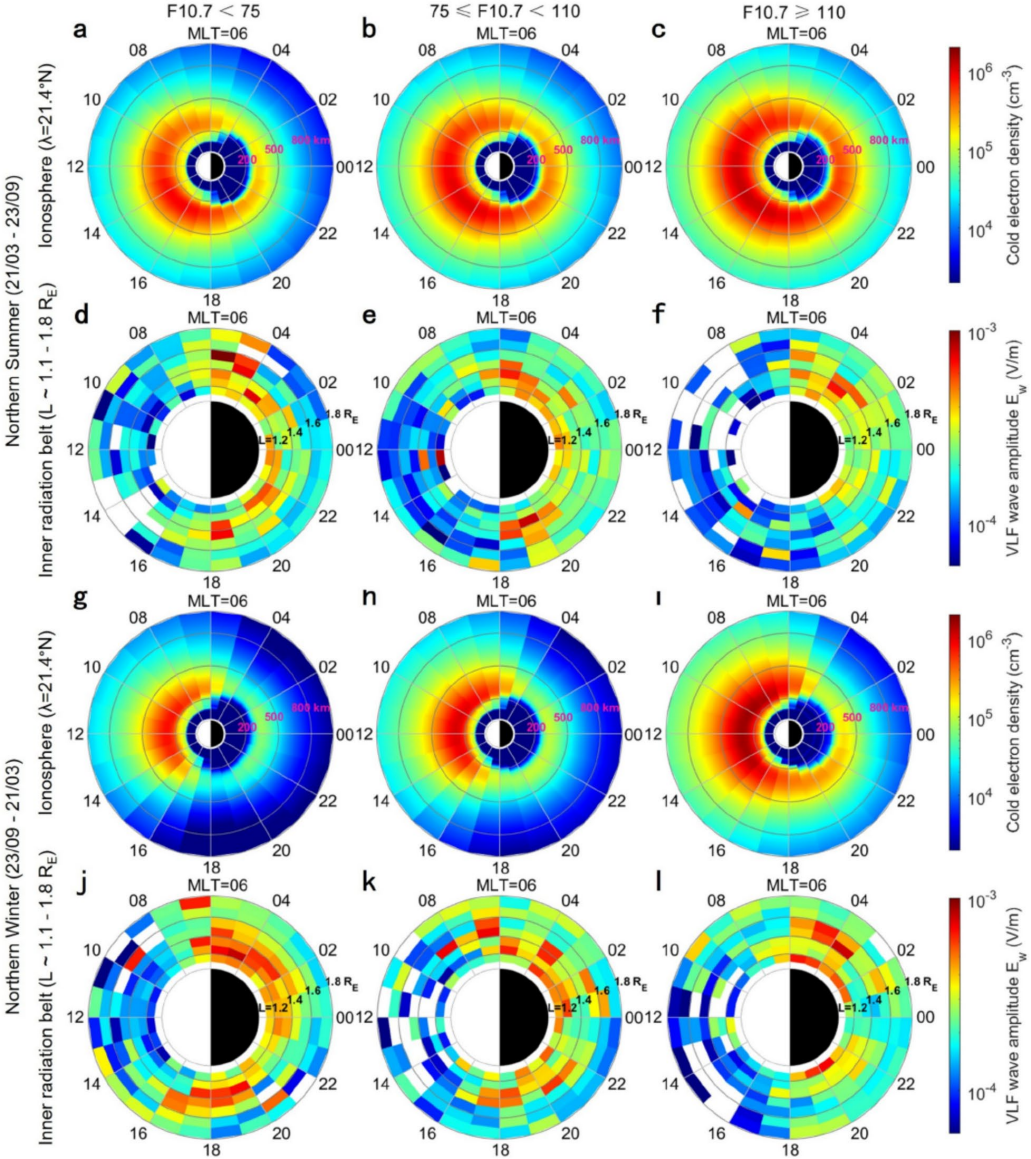
The seasonal variations of ionospheric plasma density at low latitudes of the northern hemisphere and that of artificial VLF waves radiated from the northern low-latitude transmitters (L < 1.8R_E_).

In different seasons, the intensity of artificial VLF waves in the inner belt region has also significant dependences on solar EUV radiations. Figure 4 displays the seasonal variation of ionospheric electron density at low latitudes and that of artificial VLF waves in the inner belt region. Under the same F10.7 condition, the density of ionospheric electron density at low latitudes of the northern hemisphere are higher during the northern summer (Figures 4a – c) than the northern winter (Figures 4g – i). On the contrary, the amplitudes of most VLF waves radiated from northern low-latitude transmitters are lower during the northern summer (Figures 4d – f) than the northern winter (Figures 4j – l). The seasonal variations of artificial VLF waves observed by Probe A agree with those observed by DEMETER satellite^39^.

In the same season (e.g., during the northern summer or winter), the ionospheric electron density at low latitudes also increases with the enhanced solar EUV radiations (e.g., from Figures 4a – c or 4 g – i), but the amplitudes of artificial VLF waves decrease in association with the increase in electron density (e.g., from Figures 4d – f or 4j – l). The opposite changes in the wave amplitude and plasma density suggest that the decay of VLF wave amplitudes is mainly due to the collisional damping by plasmas in low-latitude ionosphere (λ ≤ 44.2°N). In the inner radiation belt (L < 1.8R_E_), the intensity of artificial VLF waves relies strongly on that of solar EUV radiations in different seasons.

**Fig. 5:**
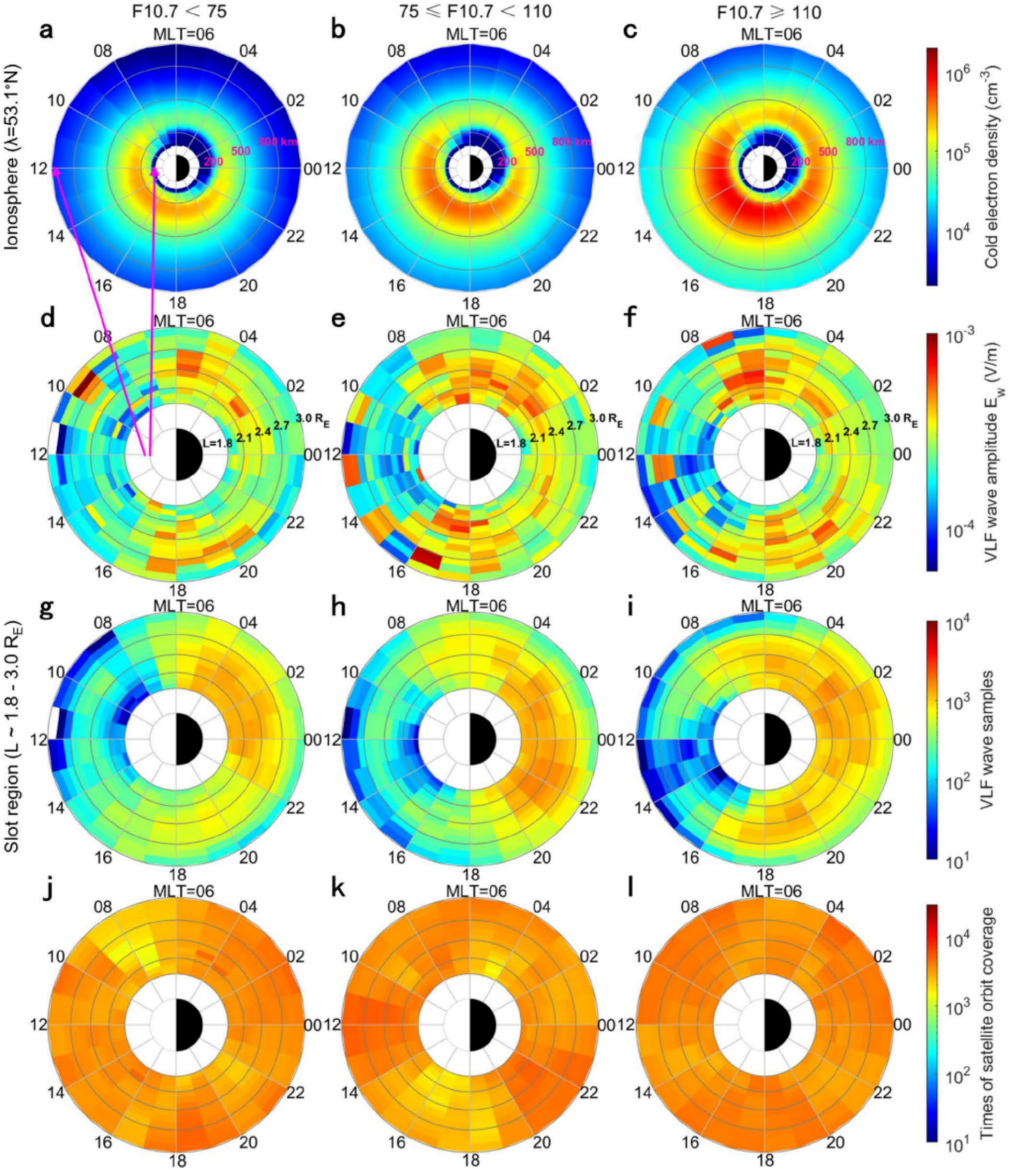
(**a**-**c**) The number density of ionospheric cold electrons at middle latitudes (λ=53.1N°) under different solar EUV radiations. (d – i) The amplitude and sample of artificial VLF waves in the slot region (L ~ 1.8–3 R_E_). (j – l) The times of satellite orbit coverage in the statistical time intervals.

The enhanced solar EUV radiations also cause slight increase in the ionospheric electron density at middle latitudes (λ = 53.1° N at the DHO38 transmitter), as indicated by Figures 5a – c. Since the middle-latitude ionosphere merely absorbs oblique solar EUV radiations, the influences of solar EUV radiations are much weaker on the middle-latitude ionosphere and VLF waves than the low-latitude ionosphere and VLF waves. Under the middle and high solar EUV radiations (F10.7 ≥ 75), the amplitude and sample of VLF waves decrease in the slot region in afternoon (MLT ~ 12–16 h), but they remain almost invariant or increase slightly at other MLTs, as indicated by Figures 5d – i. The abnormal change in the VLF waves radiated from the nightside middle-latitude transmitters is closely related to the nighttime middle-latitude trough of observed ionospheric plasma density^46,47^ (Detailed discussion in the next section). The day-night asymmetries of the middle-latitude ionosphere and VLF waves (Figures 5a - i) are weaker than those at low latitudes (Figures 3a - i).

**Fig. 6:**
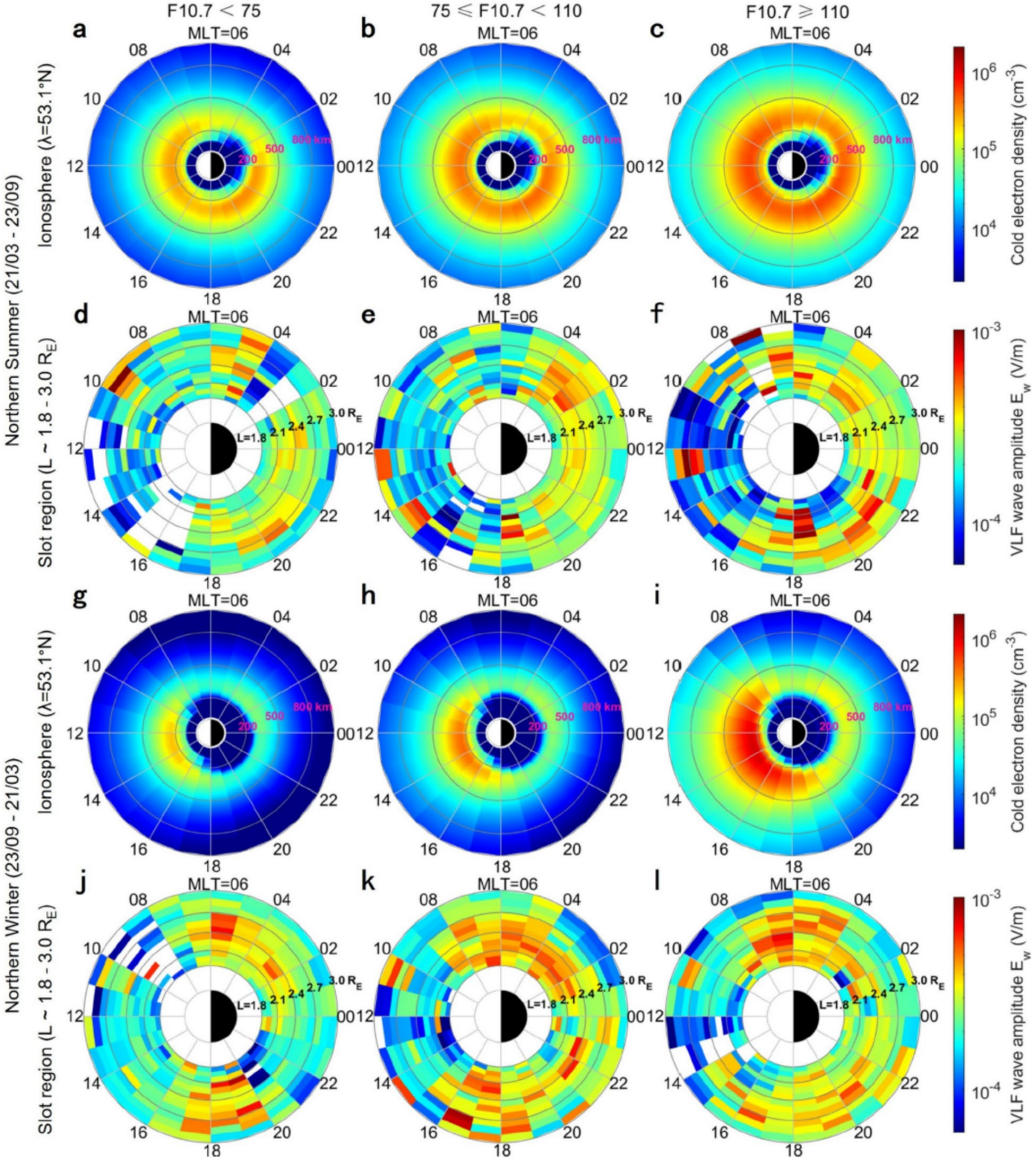
The ionospheric electron density at middle latitudes of the northern hemisphere in different seasons and the amplitude and sample of artificial VLF waves in the slot region (L > 1.8).

Interestingly, the ionospheric electron density at middle latitudes and the VLF wave amplitudes in slot region have also weak seasonal variations, as indicated by Figure 6. From the northern summer (Figures 6a – f) to winter (Figures 6g – l), the amplitudes of VLF waves radiated from the northern middle-latitude transmitters increase in association with the decrease in ionospheric electron density in the northern hemisphere. In the same season, the seasonal/semiannual averaged amplitude of artificial VLF waves around noon also decreases slightly with increasing F10.7.

Our statistical results indicate that the artificial VLF waves radiated from low-latitude transmitters (L < 1.8) have stronger responses to the enhanced solar EUV radiations than those from middle-latitude transmitters (L > 1.8). During the high EUV radiations, the larger attenuation of VLF waves at lower latitudes agrees with the full wave modeling^48,49^. Utilizing the equations 1 – 5 in the Method Section, we estimated the collision frequency of ionospheric electrons and the trans-ionospheric attenuation of artificial VLF waves under different solar EUV radiations. Since both ducted and non-ducted VLF waves can appear in the inner radiation belt and slot region^50^, their attenuations in two regions are separately evaluated. According to previous observations^10^, the wave normal angle ($\theta$) between wave vector and local magnetic field is set as $115^\circ$ and $170^\circ$ for the ducted and non-ducted VLF waves, respectively.

**Fig. 7:**
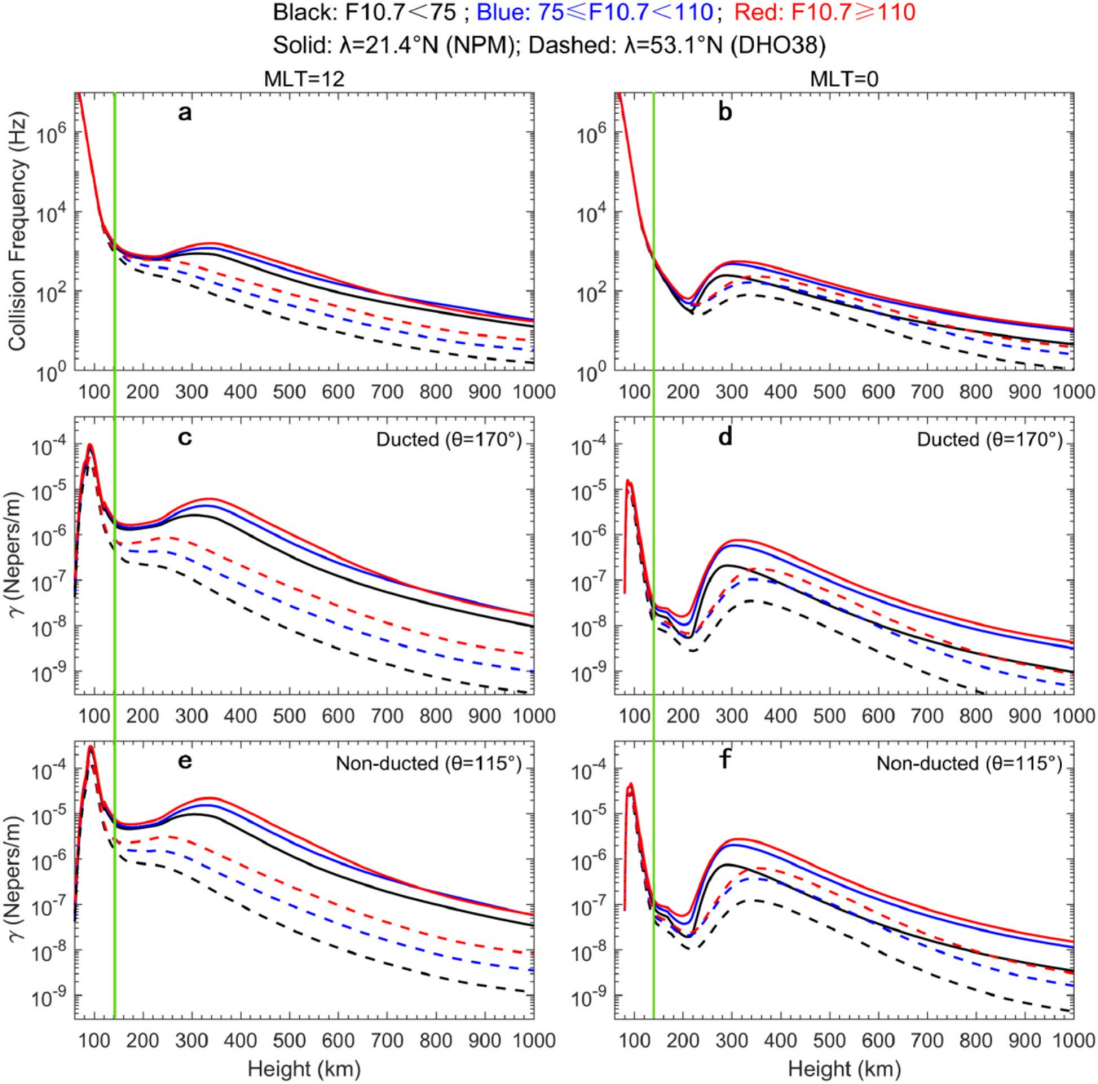
The collision frequency of ionospheric electrons and the attenuation factor ($\gamma$) of artificial VLF waves under different solar EUV radiations. (a, b) The vertical profiles of daytime and nighttime plasma collision frequencies at low and middle latitudes (λ=21.4°N and 53.1°N). (c – f) The trans-ionospheric attenuation factors of ducted and non-ducted VLF waves radiated from low- and middle-latitude transmitters. $\theta$ is the wave normal angle between wave vector and local magnetic field.

Figures 7a – 7f show the collision frequencies of daytime and nighttime electrons and the attenuation factors of ducted and non-ducted VLF waves at different latitudes, respectively. Under same F10.7 condition, the total collision frequency of ionospheric electrons in low latitudes (λ=21.4N°) is much higher than that at middle latitudes (λ=53.1N°) at MLT=12 or 00. Accordingly, the attenuation factor of artificial VLF waves from low-latitude transmitters is also larger than those from middle-latitude transmitters, as indicated by the solid and dashed curves in Figures 7c – f. At the same latitude (e.g., λ=21.4N° or 53.1N°), the attenuation of the dayside VLF waves (LT=12) is much larger than the nightside (LT=00). These calculations can account for the day-night asymmetry of the VLF waves observed by Probe A in the inner radiation belt and slot region (Figures 3 – 6).

When solar EUV radiations increase gradually from low to high levels, the total collision frequency of ionospheric electrons also increases remarkably (indicated by the black, blue and red curves in Figures 7a, b). The increased plasma collisions cause the larger attenuations of both ducted ($\theta =170^\circ$) and non-ducted ($\theta =115^\circ$) VLF waves (indicated by the black, blue and red curves in Figures 7c – f). Although the enhanced solar EUV radiations mainly increase plasma collisions in the F and topside region (> 140 km, as indicated by the green vertical line), plasma collision frequency and VLF wave attenuation remain maximum in the D and E layers (< 140 km, left of the green line). The plasma collisions in the D and E layers dominate the trans-ionospheric attenuation of artificial VLF waves under different solar EUV radiations.

**Fig. 8:**
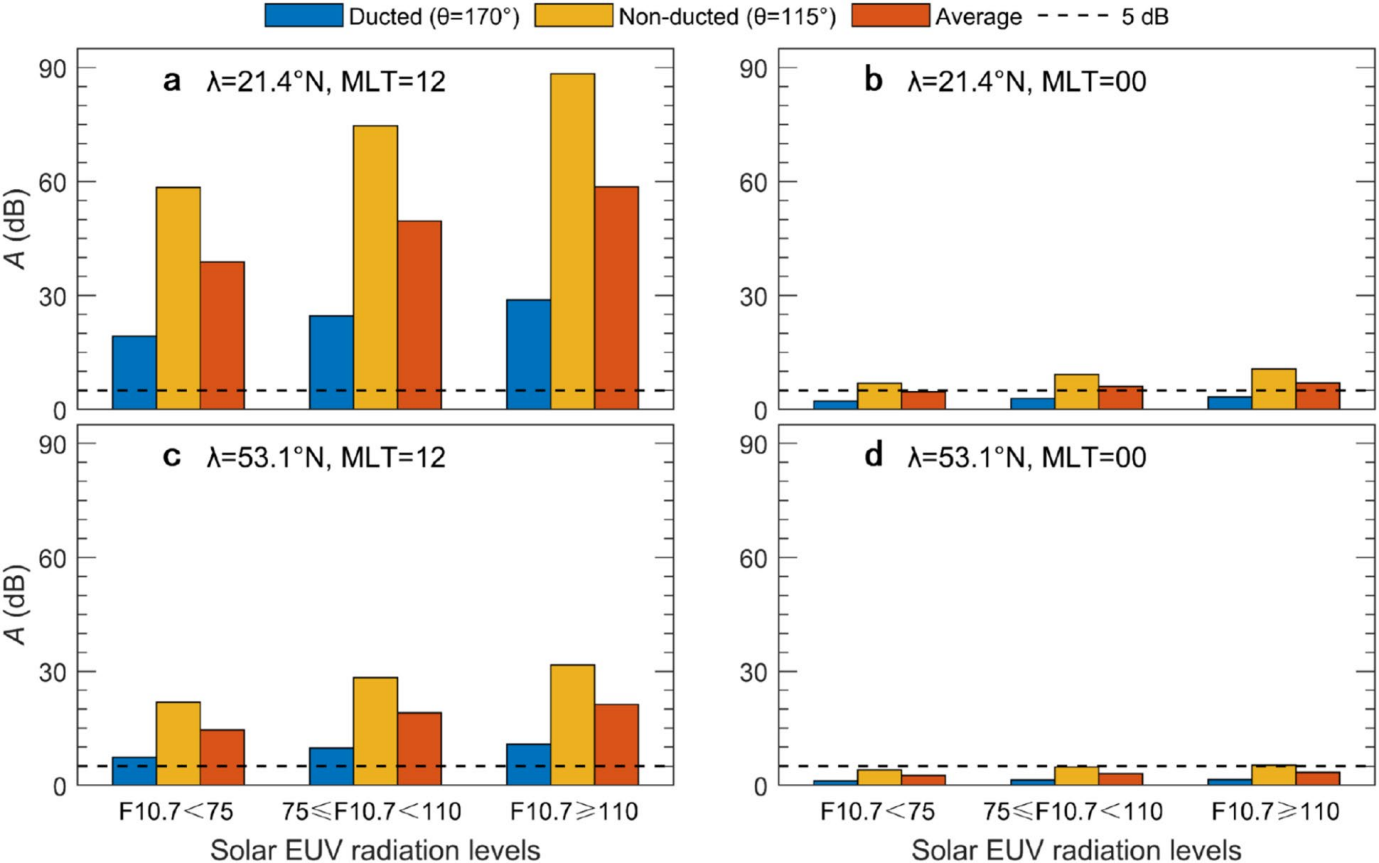
The trans-ionospheric attenuations of VLF waves from ground-based transmitters at low and middle latitudes under different solar EUV radiations. λ=21.4°N is the latitude of the NPM transmitter, and λ=53.1°N is the latitude of the DHO38 transmitter. Blue, yellow and red bars represent the attenuations of ducted and non-ducted VLF waves and their averages, respectively.

The trans-ionospheric attenuation ($A$) of artificial VLF waves is the altitude integral of attenuation factor from 60 km to 1000 km (see Equation 5 in the Method Section). Figure 8 displays the attenuations of ducted and non-ducted VLF waves and their averages when propagating through low- and middle-latitude ionospheres. Under the same F10.7 condition, the attenuations of both ducted and non-ducted VLF waves are much larger on the dayside (MLT =12h) than the nightside (MLT =00h). The larger attenuation of the dayside VLF waves reduces their amplitudes on the dayside, which can account for the day-night asymmetry of artificial VLF waves in the near-earth space. At the same MLT (e.g., MLT =12h or 00 h), the attenuation of artificial VLF waves radiated from low-latitude transmitters (λ=21.4N°) is larger than that radiated from middle-latitude transmitters (λ=53.1N°).

The attenuations of both the dayside and nightside VLF waves increase with the enhanced solar EUV radiations, indicating that the collisional damping by ionospheric plasma is enhanced. Under the middle (75 ≤ F10.7 < 110) and high (F10.7 ≥ 110) solar EUV radiations, the daytime and nighttime attenuations of ducted and non-ducted VLF waves radiated from low-latitude transmitters exceed 5 dB (Figures 8a, b). This can account for the amplitude decay of global VLF waves detected by Probe A in the inner radiation belt (Figure 3). However, the nighttime attenuation of artificial VLF waves radiated from middle-latitude transmitters (λ=53.1N°) is always less than 5 dB under different solar EUV radiations (Figure 8d), and thus their amplitudes are relatively stable in the nightside slot region (Figure 5). The attenuation of the middle-latitude VLF waves exceeds 5 dB only around noon (Figures 8a, b), and thus their amplitudes in the dayside slot region decrease slightly with the enhanced solar EUV radiations (Figure 5).

## Discussion

To validate the calculation accuracy for VLF wave attenuations, we conducted a quantitative comparison of the wave power ratios observed and calculated under different solar EUV radiations. The power ratios of the observed VLF waves are calculated via inputting the averages of VLF wave amplitudes (${E}_{\text{low}}, {E}_{\text{mid}}$ and ${E}_{\text{high}}$) in the inner radiation blet into Equations 8 and 9 in the Method Section. The theoretic power ratio is calculated via inputting the wave attenuation ($A$ in Figure 8) into Equations 10 and 11 in the Method Section. Table 2 displays the observed and calculated wave power ratios under different solar EUV radiations. The calculated value is an average for ducted and non-ducted VLF waves. The nightside calculated ratios are comparable to the observed values, but the dayside calculated ratios are less than observed values, indicating that theoretic equations (Equations 2 – 5) based on the plane wave assumption overestimate the attenuation of the dayside VLF waves. Therefore, the spherical wave propagation model^12^ or more accurate model of dayside ionosphere are required for the more accurate estimation of the VLF wave attenuation in the future.

**Table 2:** The observed and calculated power ratios of artificial VLF waves from low-latitude transmitters (L < 1.8R_E_). P_mid_/P_low_ is the power ratio of the VLF waves during the middle and low EUV radiations (F10.7 < 75, 70 ≤ F10.7 < 110), and P_high_/P_low_ is the power ratio during the high and low EUV radiations (F10.7 < 75, F10.7 ≥ 110). The calculated ratios are the averages of ducted and non-ducted VLF waves at λ=21.4°N.

Magnetic local time	MLT=12		MLT=00	
Power ratio	P_mid_/P_low_	P_high_/P_low_	P_mid_/P_low_	P_high_/P_low_
Observed values	0.6784	0.2952	0.8358	0.4195
Calculated values	0.1608	0.0557	0.7234	0.6100

Although the dayside calculations overestimate wave attenuations, the day-night difference and latitude-dependence of artificial VLF wave attenuations in theoretic calculations (Figures 7 and 8) can properly account for the satellite observations (Figures 3 – 6). The enhanced solar EUV radiations mainly increase electron collisions in low-latitude ionosphere (< 44.2°) and thus cause the significant attenuation of artificial VLF waves radiated from low-latitude transmitters (L < 1.8). The F10.7 dependence of the low-latitude VLF waves is very significant in different seasons. By contrast, the influence of solar EUV radiations on electron collisions in middle-latitude ionosphere (> 44.2°) is weaker, and thus the attenuation of artificial VLF waves radiated from middle-latitude transmitters (L > 1.8) is not significant during the high solar EUV radiations. At the same latitude (e.g., λ=21.4°N), the attenuation of artificial VLF waves is larger on the dayside than the nightside, agreeing with the Probe A observations.

Our observations and theoretical calculations demonstrate that the attenuation of artificial VLF waves radiated from lower-latitude transmitters is larger than that radiated from middle-latitude transmitters. This agrees with the previous full wave modeling^33,34^. Besides the influence of solar EUV radiations, the plasma density in middle-latitude ionosphere also relies strongly on neutral wind transports, charge exchange and recombination reactions, especially in the nighttime^42,43^. There are large-scale plasma depletions in the middle latitudes in the nighttime ionosphere^46,47^. The nighttime middle-latitude trough is helpful for the VLF wave propagation through the middle-latitude ionosphere. Thus, the amplitude of nightside VLF waves in the region of L > 1.8R_E_ does not decrease remarkably even under high solar EUV radiations. Regretfully, the nighttime middle trough of ionosphere cannot be reproduced by the IRI-2020 model, and thus its influence is not considered into our calculations. To fully understand the latitude-dependent responses of artificial VLF waves to change in solar EUV radiations, more satellite observations and more accurate ionosphere models are still required in the future.

## Methods

### Calculation of the artificial VLF wave amplitudes

Firstly, we selected the power spectral density (P_E_) of artificial VLF waves measured by Probe A under geomagnetically quiet conditions (AE < 200nT). To eliminate background noise and lightning-generated whistlers (LGW ~ 0.1–40 kHz), only the 20.05–26.74kHz waves above threshold power (P_E_ > 10^−13^ (V/m)^2^/Hz) were selected. The selected VLF waves are radiated only from ground-based transmitters in the northern hemisphere, and their amplitude (E_w_) is the square root of the integral of the wave power spectral density (P_E_) in the selected frequency range at each sample time (i), namely

1 $${E}_{w}=\sqrt{{\int }_{{f}_{\text{min}}}^{{f}_{\text{max}}}{P}_{\text{E}}\left(f\right)df{|}_{i}}$$

### Calculation of the collision frequency of ionospheric plasmas

Our statistics demonstrate that the enhanced solar EUV radiations result in the larger attenuation of artificial VLF waves from low latitudes compared to middle latitudes. The decay of VLF wave amplitudes is closely associated with the increase in the ionospheric plasma density. Under different solar EUV radiations, the collision frequency (${\nu }_{ei}$ in ${\text{s}}^{-1}$) between ionospheric electrons (e^-^) and ions (O^+^, N^+^, H^+^, He^+^, O_2_^+^, NO^+^) is expressed as^42^:

2 $${\nu }_{ei}=54.5\times \frac{{n}_{i}{Z}_{i}^{2}}{{{T}_{e}}^{3/2}}$$

where ${T}_{e}$ is the electron temperature (in kelvin), and ${n}_{i}$ is the ion density (in cm^−3^), and Z_i_ is the charge number of ion species (i). ${T}_{e}$ and ${n}_{i}$ are calculated with the IRI-2020 model.

The elastic collision frequency (${\nu }_{en}$) between ionospheric electrons (e^-^) and neutral atmospheric compositions (N_2_, O_2_, O, He, and H) are listed in Table 3. $n\left({\text{N}}_{2}\right)$, $n\left({\text{O}}_{2}\right)$, $n\left(\text{O}\right)$. $n\left(\text{He}\right)$ and $n\left(\text{H}\right)$ are the neutral densities in the Earth’s atmosphere, and they are calculated with the NRLMSIS 2.0 atmospheric model^51^. The total collision frequency of ionospheric electrons is:

**Table 3:** Elastic collision frequency (${\nu }_{en})$ between electrons and neutral atmospheric compositions^42^.

Species	${\nu }_{en} ({\text{s}}^{-1}$)
N_2_	$2.33\times {10}^{-11}n\left({\text{N}}_{2}\right)\left(1-1.21\times {10}^{-4}{T}_{e}\right){T}_{e}$
O_2_	$1.82\times {10}^{-10}n\left({\text{O}}_{2}\right)\left(1+3.6\times {10}^{-2}{{T}_{e}}^{1/2}\right){{T}_{e}}^{1/2}$
O	$8.9\times {10}^{-11}n\left(\text{O}\right)\left(1+5.7\times {10}^{-4}{T}_{e}\right){{T}_{e}}^{1/2}$
He	$4.6\times {10}^{-10}n\left(\text{He}\right){{T}_{e}}^{1/2}$
H	$4.5\times {10}^{-9}n\left(\text{H}\right)\left(1-1.35\times {10}^{-4}{T}_{e}\right){T}_{e}^{1/2}$

3 $$\nu =\sum_{i}{\nu }_{ei}+\sum_{n}{\nu }_{en}$$

### Calculation of the artificial VLF wave attenuations

The collisional damping of the ionospheric electrons is still effective for whistler-mode VLF waves even though the collision frequency of electrons is lower than the wave frequency^52^. The attenuation factor ($\gamma$ in Nepers/m) of the VLF waves is expressed as^39^:

4 $$\gamma =\frac{\omega }{\sqrt{2}c}{\left[\sqrt{{(1+G\left|{Y}_{L}\right|)}^{2}+{(GZ)}^{2}}-(1+G\left|{Y}_{L}\right|)\right]}^\frac{1}{2}$$

where $G=\frac{X}{{{Y}_{L}}^{2}+{Z}^{2}}$. $X$ equals to the squared plasma frequency divided by the squared wave frequency. ${Y}_{L}=cos\left(\theta \right)Y$, and $\theta$ is the wave normal angle, and $Y$ equals to the cyclotron frequency divided by the wave frequency. Z equals to the collision frequency ($v$) divided by wave angular frequency.

The trans-ionospheric attenuation (*A* in dB) of ground-based VLF transmitter waves is an altitude integral of attenuation factor from the bottom (*h*_0_) to topside (h_1_) of ionosphere^39^:

5 $$A=8.69\times {10}^{3}{\int }_{{h}_{0}}^{{h}_{1}}\gamma dh$$

where, *h*_0_=60 km, and *h*_1_=1000 km.

### Ratio of the VLF wave powers under different solar EUV radiations

The power of electromagnetic waves is proportional to the square of its amplitude. Therefore, the ratios of artificial VLF wave powers under different solar EUV radiations are respectively expressed as:

6 $$\frac{{P}_{\text{mid}}}{{P}_{\text{low}}}=\frac{{E}_{\text{mid}}^{2}}{{E}_{\text{low}}^{2}}$$

7 $$\frac{{P}_{\text{high}}}{{P}_{\text{low}}}=\frac{{E}_{\text{high}}^{2}}{{E}_{\text{low}}^{2}}$$

where ${E}_{\text{low}}, {E}_{\text{mid}}$ and ${E}_{\text{high}}$ are the VLF wave amplitudes observed by Probe A under the low, middle and high levels of solar EUV radiations, respectively.

In theory, the wave attenuation A (dB) is the functions of its powers at 60 km [${P}_{60}$] and 1000 km [${P}_{1000}$] for a given F10.7 condition. Thus, the change in A under different F10.7 conditions is expressed:

8 $$A_{{{\text{mid}}}} - { }A_{{{\text{low}}}} = 10{\text{log}}_{10} (\frac{{{\text{P}}_{{{\text{low}}}} }}{{{\text{P}}_{{{\text{mid}}}} }})$$

9 $$A_{high} - { }A_{low} = 10log_{10} (\frac{{P_{{{\text{low}}}} }}{{P_{{{\text{mid}}}} }})$$

From equations (8) and (9), we can get:

10 $$\frac{{P}_{\text{mid}}}{{\text{P}}_{\text{low}}}={10}^{-(\frac{{A}_{\text{mid}}- {A}_{\text{low}}}{10})}$$

11 $$\frac{{P}_{\text{high}}}{{P}_{\text{low}}}={10}^{-(\frac{{A}_{\text{high}}- {A}_{\text{low}}}{10})}$$

## Data Availability

The EMFISIS data of Van Allen Probes are publicly available from https://emfisis.physics.uiowa.edu/data/index. F10.7 and AE index are available from http://cdaweb.gsfc.nasa.gov/sp_phys. The IRI-2020 model is available from https://kauai.ccmc.gsfc.nasa.gov/instantrun/iri/. The NRLMSIS atmospheric model is publicly available at the Web https://kauai.ccmc.gsfc.nasa.gov/instantrun/nrlmsis/. The statistical and calculated data are publicly available at 10.6084/m9.figshare.28225754.v4.
